# Engineering TnpB as a compact RNA-guided genome editor from molecular constraints to design principles

**DOI:** 10.3389/fgeed.2026.1945303

**Published:** 2026-09-22

**Authors:** Bin Ma, Yimiao Li, Dongsheng Zhang, Yu Wu, Jie Gao, He Li, Shuang Jia

**Affiliations:** 1 Key Laboratory of Herbage and Endemic Crop Biology, Ministry of Education, School of Life Sciences, Inner Mongolia University, Hohhot, Inner Mongolia, China; 2 School of Automation and Electrical Engineering, Inner Mongolia University of Science and Technology, Baotou, China

**Keywords:** compact genome editor, genome editing, RNA-guided endonuclease, TnpB, transposon-associated motif, ωRNA/reRNA scaffold

## Abstract

Recent discoveries of non-CRISPR RNA-guided nucleases have expanded the landscape of programmable genome editing beyond canonical Cas systems. Among these, TnpB proteins derived from IS200/IS605-family transposable elements are ultra-compact RNA-guided DNA endonucleases with potential for delivery-constrained genome engineering. Here, we review the structural and mechanistic basis of TnpB function, including protein architecture, transposon-associated motif (TAM) recognition, ωRNA/reRNA scaffold organization, RNA–DNA heteroduplex formation, and RuvC-mediated DNA cleavage. We highlight that TnpB activity emerges from coordinated interactions between the nuclease and its RNA scaffold, which jointly influence ribonucleoprotein assembly, target recognition, catalytic activation, and specificity. We further discuss engineering strategies involving ortholog selection, protein and TAM-recognition engineering, RNA scaffold optimization, delivery, and specificity control, and relate these approaches to current applications across biological systems. Although TnpB remains less mature than established CRISPR-Cas platforms, its compact architecture and growing engineering toolkit support its development as a complementary genome editing platform. Future progress will benefit from establishing predictive models that integrate target accessibility, RNA–protein compatibility, activity, and fidelity.

## 1 Introduction

Genome editing technologies have transformed biotechnology by enabling programmable manipulation of genetic information across diverse biological systems, including functional genomics, synthetic biology, agriculture, and biomedical research. RNA-guided nucleases have become central tools in this field because they combine sequence-specific targeting with relatively simple guide-based programmability. Among these systems, CRISPR-Cas nucleases, particularly Cas9 and Cas12, have achieved widespread adoption owing to their robust activity, extensive engineering history, and broad range of derivative applications, including gene disruption, base editing, prime editing, and transcriptional regulation (Jinek et al., 2012; Cong et al., 2013; Zetsche et al., 2015; Komor et al., 2016; Gaudelli et al., 2017; Anzalone et al., 2019). However, the expansion of genome editing toward therapeutic delivery, agricultural biotechnology, and synthetic biology has highlighted several limitations of conventional editors, including large protein size, restricted delivery capacity, target-site accessibility constraints, and context-dependent editing performance (Fu et al., 2013; Hsu et al., 2013; Ran et al., 2015; Pickar-Oliver and Gersbach, 2019).

These limitations have motivated the search for smaller RNA-guided nucleases that can overcome the packaging constraints of current editing systems. Recent studies have revealed that programmable RNA-guided DNA targeting is not restricted to CRISPR-associated immune systems but is also present in diverse mobile genetic elements. Among these emerging systems, the transposon-associated RNA-guided DNA endonuclease TnpB proteins encoded by IS200/IS605-family transposable elements represent a distinct class of compact RNA-guided DNA endonucleases. Evolutionary and structural analyses have demonstrated a close relationship between TnpB and Cas12-family nucleases, suggesting that mobile genetic elements represent an important reservoir of programmable nuclease diversity beyond canonical CRISPR systems (Altae-Tran et al., 2021; Karvelis et al., 2021; Altae-Tran et al., 2023).

A defining feature of TnpB is its exceptionally compact architecture. Most characterized TnpB proteins are approximately 350–400 amino acids in length, substantially smaller than commonly used genome editing nucleases such as *Streptococcus pyogenes* Cas9 and many Cas12 orthologs (Karvelis et al., 2021; Sasnauskas et al., 2023). This compactness provides potential advantages for applications where delivery capacity represents a major constraint, including viral vector-based delivery, compact genetic circuits, and multiplex genome engineering. Mechanistically, TnpB-mediated DNA targeting requires coordinated interaction among three essential components: the TnpB protein, a structured transposon-derived ωRNA/reRNA scaffold, and a target DNA sequence containing a compatible transposon-associated motif (TAM). This RNA scaffold is commonly referred to as ωRNA (omega RNA) or reRNA (right-end RNA), reflecting its origin from the transposon-associated RNA architecture. The term ωRNA is frequently used to describe the TnpB-associated RNA scaffold in structural and evolutionary studies, whereas reRNA emphasizes its origin from the right end of IS200/IS605-family transposons and is commonly used in mechanistic and genome-editing studies. Therefore, ωRNA and reRNA generally refer to the same TnpB-associated RNA scaffold, although the preferred terminology varies depending on the research context. Unlike Cas9 sgRNA or Cas12a crRNA, which mainly provide target recognition functions, TnpB-associated ωRNA/reRNA contains additional structural elements required for ribonucleoprotein assembly and nuclease activation, reflecting a greater contribution of RNA architecture to TnpB function rather than a simple compensation for reduced protein size. Structural studies have revealed that the TnpB protein contains a compact nuclease architecture composed of recognition-associated domains and a RuvC-like catalytic domain, while the RNA scaffold contributes to ribonucleoprotein assembly and guide-directed RNA–DNA heteroduplex formation (Sasnauskas et al., 2023; Nakagawa et al., 2023). Typically, Cas9 sgRNAs are approximately 100 nucleotides in length, whereas Cas12a crRNAs are approximately 40–50 nucleotides. In comparison, TnpB ωRNA/reRNA scaffolds are often larger (approximately 150–200 nucleotides in many characterized systems, although their length varies among orthologs and engineered scaffold designs), reflecting their dual roles in target guidance and structural organization of the TnpB ribonucleoprotein complex.

Despite these advantages, TnpB remains an emerging genome editing platform rather than a mature alternative to established CRISPR systems. Unlike Cas9 and Cas12 nucleases, which benefit from extensive engineering, standardized guide design principles, and well-characterized specificity profiles, TnpB performance remains highly variable among orthologs, target sites, RNA scaffolds, and cellular contexts. Current limitations include ortholog-specific TAM requirements, incomplete understanding of ωRNA/reRNA design principles, variable editing efficiency, limited mismatch tolerance characterization, and insufficient genome-wide specificity assessment (Karvelis et al., 2021; Feng et al., 2024; Marquart et al., 2024).

Accumulating structural and engineering evidence indicates that TnpB activity is determined by coordinated interactions between protein and RNA components rather than by nuclease sequence alone. The TnpB protein contributes to TAM recognition, conformational activation, and catalytic competence, whereas the ωRNA/reRNA scaffold regulates ribonucleoprotein assembly, guide positioning, and productive target engagement (Sasnauskas et al., 2023; Nakagawa et al., 2023; Marquart et al., 2024). Based on these observations, we propose a coupled protein–RNA constraint framework in which editing performance emerges from the compatibility between nuclease architecture and RNA scaffold configuration. This framework provides a conceptual basis for understanding the heterogeneous activity of natural TnpB variants and highlights the need for coordinated engineering of both protein and RNA components.

Thus, the key challenge is not merely discovering smaller nucleases, but determining whether compact editors such as TnpB can achieve sufficient activity, target flexibility, and specificity in different biological systems. Addressing this challenge requires integrated optimization strategies, including ortholog discovery, protein engineering, TAM compatibility expansion, RNA scaffold redesign, delivery optimization, and systematic specificity evaluation (Marquart et al., 2024; Wang et al., 2024). Although protein–RNA coordination is a general feature of RNA-guided nucleases, we focus on the ortholog-specific coupled constraints that shape TnpB engineering. The distinctive emphasis of this review is to connect molecular mechanism with engineering outcomes through a coupled-constraint framework, integrating natural ortholog diversity, protein and RNA engineering, delivery, specificity, and cross-system performance into a unified set of design principles.

We first summarize the molecular mechanisms underlying TnpB-mediated genome editing and compare TnpB with other RNA-guided nuclease platforms, followed by discussions of current engineering strategies, applications, and future perspectives. We further discuss future opportunities and challenges for developing TnpB as a complementary class of compact genome editing tools for biotechnology applications.

## 2 Molecular basis of TnpB

Transposition plays a fundamental role in shaping the genomes of all life forms. Insertion sequences (IS), particularly the IS200/IS605 family, represent a class of relatively simple mobile genetic elements that encode only the core components required for their mobility and regulation (Siguier et al., 2014; Ton-Hoang et al., 2005). These elements typically encode the TnpA transposase, which is essential for transposition, and in many cases also an accessory protein, TnpB. Unlike TnpA, TnpB is not required for transposition, suggesting that it may have evolved an independent functional role beyond canonical mobile element propagation (Karvelis et al., 2021). Recent studies have identified TnpB as a compact RNA-guided DNA endonuclease associated with IS200/IS605 elements. Its activity depends on a RuvC-like nuclease core, a transposon-encoded ωRNA/reRNA guide, and a target DNA sequence containing a compatible transposon-associated motif (TAM). This molecular organization not only provides insight into the functional diversification of transposon-associated proteins, but also offers a basis for understanding the evolutionary relationship between TnpB and CRISPR-associated nucleases such as Cas12, as well as for developing TnpB as a programmable genome editing platform (Karvelis et al., 2021; Sasnauskas et al., 2023; Altae-Tran et al., 2023).

### 2.1 Overall structures of TnpB complexes

Structural characterization of TnpB has been centered primarily on the ISDra2 TnpB ortholog from *Deinococcus radiodurans*, a 408-amino-acid RNA-guided nuclease that has served as the principal model for defining the architecture of the TnpB ribonucleoprotein complex (Figure 1B1). Two independent cryo-electron microscopy (cryo-EM) studies provided complementary structural views of this system. Sasnauskas et al. resolved a DNA-free TnpB–reRNA binary complex at 2.8 Å and two DNA-bound TnpB–reRNA–DNA ternary conformations at 3.14 and 2.96 Å, respectively (PDB 8BF8, 8EXA and 8EX9). Independently, Nakagawa et al. determined a 3.2-Å cryo-EM structure of the ISDra2 TnpB–ωRNA–target DNA ternary complex. Together, these structures establish the molecular architecture of the preassembled TnpB ribonucleoprotein (RNP) and provide snapshots of structural transitions associated with target DNA engagement (Sasnauskas et al., 2023; Nakagawa et al., 2023).

**FIGURE 1:**
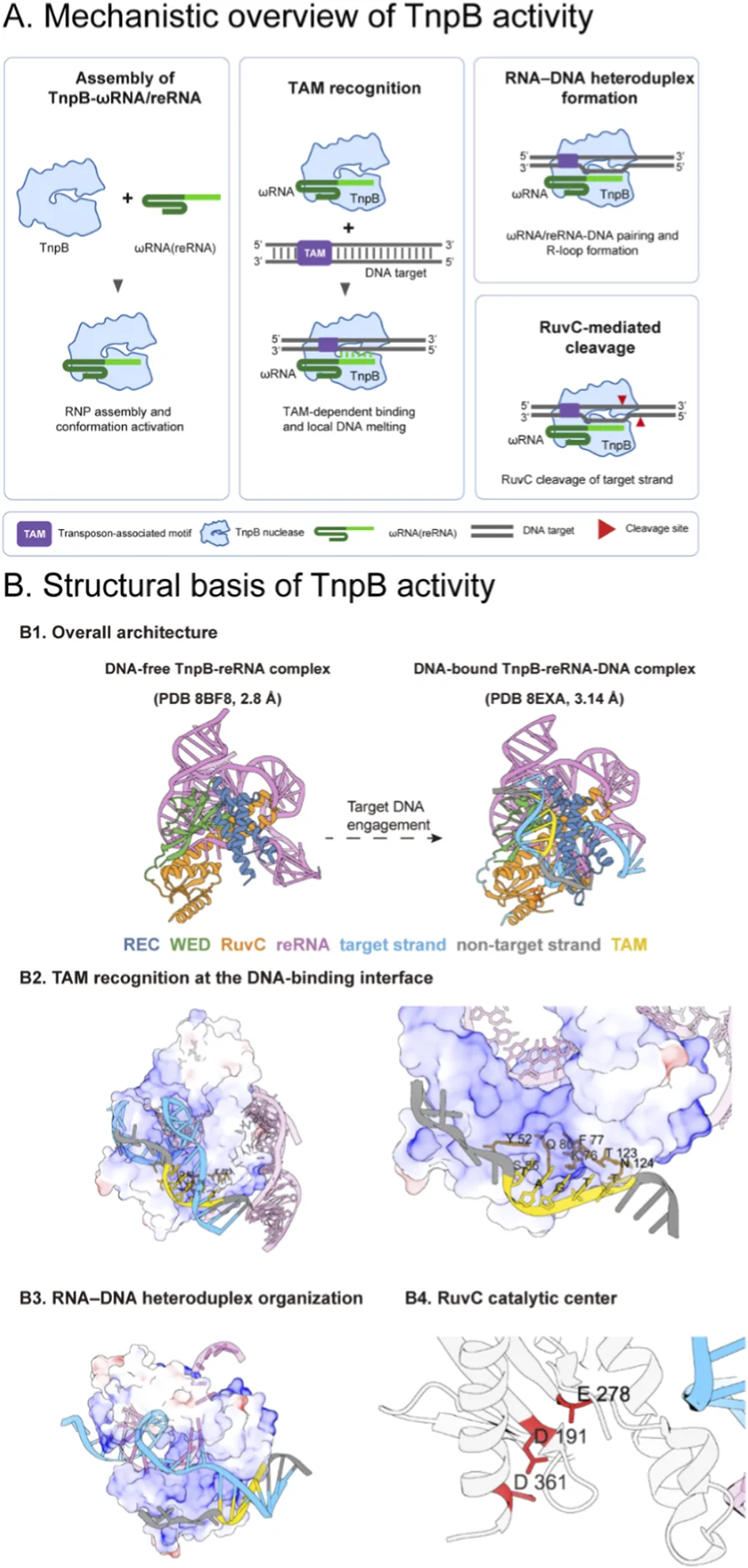
Molecular basis of TnpB activity. **(A)** Mechanistic overview of TnpB-mediated genome targeting, including ribonucleoprotein assembly, TAM recognition, RNA–DNA heteroduplex formation, and RuvC-mediated cleavage. **(B)** Structural basis underlying TnpB activity, including overall architecture, TAM recognition interface, RNA–DNA heteroduplex organization, and catalytic center.

ISDra2 TnpB adopts a compact bilobed architecture composed of recognition (REC) and nuclease (NUC) lobes. The REC lobe contains the wedge (WED) and REC domains, whereas the NUC lobe contains the RuvC catalytic domain and a target-nucleic-acid-binding (TNB) domain; a C-terminal region is poorly resolved in the available ternary structure. The structured ωRNA/reRNA scaffold is accommodated mainly along the WED and RuvC surfaces, whereas the guide RNA–target DNA heteroduplex occupies a positively charged central channel formed by the WED, REC and RuvC domains. The TAM-containing DNA duplex is positioned at the WED–REC interface. This organization places RNA scaffold recognition, TAM sensing, heteroduplex accommodation and RuvC-mediated catalysis within a single compact structural framework rather than in extensively separated functional modules (Nakagawa et al., 2023).

Importantly, currently available structures do not define the conformational transition associated with initial ωRNA/reRNA loading because an experimentally determined RNA-free apo TnpB structure has not yet been reported. Instead, the most informative comparison is between the DNA-free TnpB–reRNA binary complex and DNA-bound ternary complexes. Sasnauskas et al. captured two DNA-bound conformations that differ particularly in the organization of the RuvC region, which is resolved in ternary conformation one but poorly resolved in conformation 2, indicating conformational heterogeneity of the catalytic region during target engagement. Structural and biochemical analyses further support a model in which RNA–DNA heteroduplex formation displaces the distal portion of reRNA stem two and relieves its constraint on the RuvC region, thereby coupling R-loop formation to acquisition of a cleavage-competent state (Sasnauskas et al., 2023). Consistent with this model, subsequent ribonucleoprotein mutational scanning identified activity-enhancing substitutions and deletions concentrated around a hinge region in reRNA stem 2, although the precise structural mechanism by which these changes promote TnpB activation remains unresolved (Thornton et al., 2026).

Comparison with more extensively characterized CRISPR nucleases highlights both conserved principles and important gaps in the current TnpB structural framework. In Cas9, guide-RNA loading induces extensive reorientation of the two major structural lobes to form a central channel competent for target DNA binding, demonstrating a prominent RNA-induced activation step before DNA engagement (Jinek et al., 2014). In Cas12a, progressive crRNA–DNA heteroduplex formation triggers conformational rearrangements that open the catalytic cleft and activate the RuvC nuclease center (Stella et al., 2018). By contrast, available TnpB structures primarily describe conformational changes occurring within an already assembled TnpB–reRNA complex during target DNA engagement. Because neither an RNA-free apo TnpB structure nor a complete series of target-recognition intermediates is currently available, it remains premature to determine whether the overall conformational trajectory of TnpB is intrinsically more restricted than that of Cas9 or Cas12a. Resolving these missing states will therefore be important for defining how the compact architecture of TnpB coordinates RNA loading, target recognition and catalytic activation.

### 2.2 TAM-dependent target recognition

Target DNA recognition by TnpB is initiated through binding of a transposon-associated motif (TAM), which acts as an initial recognition determinant for productive guide–target pairing and subsequent DNA cleavage. In ISDra2 TnpB, the cognate 5′-TTGAT-3′ TAM is accommodated in a cleft formed primarily by the REC and WED domains, immediately adjacent to the guide RNA–target DNA heteroduplex. Structural analysis revealed that TnpB recognizes the TAM through a network of base-specific and van der Waals contacts involving both DNA strands, thereby coupling motif discrimination directly to the architecture of the target-binding channel (Nakagawa et al., 2023).

At the residue level, the −1 thymine of the non-target strand interacts with Y52 and S56 in the REC domain, whereas the −2 adenine forms a hydrogen bond with Q80. The −3 guanine is recognized through a bidentate hydrogen-bonding interaction with K76, whose side chain is stabilized by F77. F77 also forms van der Waals contacts with dT18 in the target strand and, together with T123 in the WED domain, contributes to recognition of the −4 thymine, whereas N124 forms a hydrogen bond with the −5 thymine. Consistent with these structural contacts, alanine substitutions at Y52, K76, Q80 or T123 abolished DNA cleavage under the conditions tested, whereas S56A, F77A and N124A substantially reduced cleavage activity (Nakagawa et al., 2023). These findings define a compact TAM-recognition interface distributed across the REC and WED domains rather than a single isolated motif-recognition module.

TAM recognition is mechanistically coupled to subsequent target engagement. The TAM-containing duplex is positioned adjacent to the channel accommodating the guide RNA–target DNA heteroduplex, placing motif recognition upstream of local DNA opening and R-loop formation. Structural analyses of DNA-free and DNA-bound ISDra2 TnpB complexes support a sequential model in which TAM recognition promotes productive target engagement and facilitates the initiation of guide–target pairing, thereby establishing the nucleic-acid configuration required for subsequent R-loop propagation and catalytic activation (Nakagawa et al., 2023; Sasnauskas et al., 2023).

Importantly, TAM requirements vary substantially among TnpB orthologs. Comparative characterization of diverse IS605-associated TnpB systems has revealed considerable variation in TAM preference, indicating that target-site accessibility is strongly dependent on the specific TnpB ortholog examined (Xiang et al., 2024). For example, SisTnpB7 from *Sulfolobus islandicus* preferentially recognizes the cognate 5′-TTTAA-3′ TAM but can also support genome editing at multiple weaker TAM variants. Systematic profiling showed relatively relaxed sequence requirements at several TAM positions, and weaker TAMs such as CTTGA and ATTGA supported efficient targeted gene deletion at selected genomic loci (Feng et al., 2024). This natural diversity expands the potential targeting space of the TnpB family but also emphasizes that TAM compatibility and activity must be characterized for individual orthologs rather than inferred from a universal TnpB recognition rule.

The structurally defined TAM-contacting residues also provide direct molecular targets for engineering target recognition. Indeed, subsequent modification of the ISDra2 TnpB TAM interface demonstrated that substitutions at K76 can alter TAM preference and expand recognition of alternative motifs (Marquart et al., 2024), as discussed in Section 4.2 (Figure 1B2).

### 2.3 RNA–DNA heteroduplex formation and DNA unwinding

Following TAM-dependent target engagement, TnpB promotes guide–target pairing and formation of an RNA–DNA heteroduplex, accompanied by displacement of the non-target DNA strand to generate an R-loop (Figure 1B3). In ISDra2 TnpB, the 16-nt guide sequence pairs with the target strand within a positively charged channel formed primarily by the REC and RuvC domains. At the TAM-proximal end, the phosphate backbone between target-strand nucleotides dA17 and dC16 is contacted by Q121 and N156 in the WED domain, whereas the first guide–target base pair is stabilized by residues in the WED and RuvC domains. These interactions facilitate initial target DNA unwinding and position the nascent RNA–DNA heteroduplex within the central nucleic-acid-binding channel (Nakagawa et al., 2023).

Recognition of the heteroduplex is strongly position dependent. Structural analysis showed that guide–target base pairs 1–11 in the TAM-proximal region are accommodated within the central channel and recognized predominantly through interactions with the sugar–phosphate backbone. In contrast, the 12th base pair is exposed to solvent, whereas the four terminal base pairs at positions 13–16 are disordered in the available structure. Consistent with these structural features, double mismatches introduced within guide positions 1–12 abolished detectable cleavage in biochemical assays, whereas mismatches at positions 13–16 reduced but did not eliminate cleavage. Genome-wide profiling in human cells likewise identified off-target sites containing mismatches preferentially in the TAM-distal region. These observations indicate that approximately the first 12 TAM-proximal base pairs constitute the principal specificity-determining region of the ISDra2 TnpB heteroduplex, whereas distal pairing is subject to less extensive protein surveillance (Nakagawa et al., 2023).

Heteroduplex formation also imposes an energetic requirement for DNA unwinding that can limit productive target engagement. In the ISDra2 ternary structure, the TAM-distal end of the heteroduplex and the re-hybridized DNA duplex are disordered and located away from the TNB domain. Biochemical experiments further showed that ISDra2 TnpB cleaved substrates containing AT-rich sequences in the re-hybridized region more efficiently than substrates containing GC-rich sequences. On this basis, Nakagawa et al. proposed that, unlike the Cas12 enzymes examined structurally, ISDra2 TnpB has limited capacity to actively unwind the re-hybridized distal DNA duplex and instead relies more strongly on spontaneous DNA opening to make the substrate accessible to the RuvC active site (Nakagawa et al., 2023). This mechanism provides a potential explanation for why local duplex stability can influence TnpB cleavage independently of guide–target complementarity alone.

A recent study examining *Youngiibacter multivorans* TnpB (Ymu1 TnpB) further revealed that DNA unwinding proceeds through discrete energetic states. Using biochemical assays and gold rotor-bead tracking, Zhou et al. observed transitions from a closed state to a relatively long-lived partially unwound intermediate and subsequently to a fully unwound open state. Wild-type (WT) Ymu1 TnpB predominantly occupied the closed state, frequently reverted from the intermediate state, and only rarely formed a stable open complex under torsionally relaxed conditions. Mutational analysis identified distinct determinants of these transitions: the WED-domain substitution H4W primarily stabilized the intermediate state, whereas combining H4W with the lid-adjacent substitutions L304F and V305R generated the Ymu1-WFR variant, which stabilized both intermediate and open states and markedly enhanced DNA cleavage and genome-editing activity (Zhou et al., 2026).

Together, these observations identify DNA unwinding as a molecular checkpoint in productive TnpB target recognition rather than merely a passive consequence of guide–target complementarity. The ISDra2 structural and biochemical data and the Ymu1 single-molecule measurements indicate that R-loop formation is constrained by the energetic cost of duplex opening, although the capacity to stabilize specific unwinding intermediates may differ among TnpB orthologs. In this framework, TAM recognition initiates target engagement, progressive guide–target pairing drives R-loop formation, and stabilization of sufficiently unwound DNA states promotes formation of a cleavage-competent complex. These findings also provide a mechanistic rationale for engineering the DNA-unwinding energy landscape to enhance TnpB activity, while the subsequent positioning and cleavage of DNA within the RuvC catalytic center are discussed in Section 2.4.

### 2.4 RuvC-mediated catalytic activation and DNA cleavage

TnpB-mediated DNA cleavage is catalyzed by a single RuvC nuclease center located within the NUC lobe. In ISDra2 TnpB, the RuvC domain adopts an RNase H-like fold and contains the conserved acidic residues D191, E278 and D361, which form a catalytic center structurally analogous to those of Cas12-family nucleases (Nakagawa et al., 2023; Sasnauskas et al., 2023). Functional analyses further established the importance of this catalytic core: the D191A substitution strongly compromised target DNA cleavage *in vitro*, whereas D191A and E278A variants impaired plasmid interference in *Escherichia coli* (Karvelis et al., 2021). Together, these structural and biochemical data establish the RuvC domain as the catalytic center responsible for TnpB-mediated double-stranded DNA cleavage (Figure 1B4).

Unlike Cas9, which uses spatially distinct HNH and RuvC nuclease domains to cleave the target and non-target strands, respectively, TnpB uses a single RuvC active site to process both DNA strands, similar to Cas12 nucleases. For ISDra2 TnpB, cleavage mapping showed that the target strand is cleaved approximately 21 nucleotides downstream of the TAM, whereas cleavage of the non-target strand occurs at multiple positions approximately 15–21 nucleotides downstream of the TAM, generating staggered double-strand breaks with 5′ overhangs (Karvelis et al., 2021; Nakagawa et al., 2023). Although both strands are processed by the same catalytic center, the complete sequence of strand loading and cleavage has not yet been structurally resolved in TnpB.

Catalytic activation is coupled to formation of the RNA–DNA heteroduplex rather than occurring constitutively after TAM recognition. Structural analysis identified a lid-like subdomain adjacent to the RuvC center that sterically occludes access to the catalytic site and contacts the minor-groove side of the RNA–DNA heteroduplex around guide–target base pairs 7–8 (Sasnauskas et al., 2023). This arrangement provides a structural mechanism through which progression of guide–target pairing can be communicated to the nuclease center. Consistent with a gating role for this region, subsequent mutational scanning identified activity-enhancing substitutions at P282, which lies near the boundary of the RuvC lid. Hydrophobic substitutions were enriched at this position, leading Thornton et al. to propose that altered lid flexibility may facilitate the conformational transition associated with TnpB activation (Thornton et al., 2026). However, intermediate structures directly defining lid opening and substrate entry into the RuvC center remain unavailable.

Substrate delivery to the RuvC active site also highlights a mechanistic difference between ISDra2 TnpB and structurally characterized Cas12 effectors. As discussed in Section 2.3, the TNB domain of ISDra2 TnpB does not substantially engage the TAM-distal heteroduplex or re-hybridized DNA duplex. Consequently, ISDra2 TnpB appears to rely more strongly on spontaneous DNA opening to make the cleavage substrate accessible to the RuvC center. By contrast, structurally characterized Cas12 enzymes use additional interactions with the distal heteroduplex and re-hybridized DNA to promote further unwinding and facilitate strand loading into the RuvC active site. Thus, although TnpB and Cas12 share a single-RuvC catalytic architecture, their mechanisms of generating and delivering cleavage substrates to the active site differ in important respects (Nakagawa et al., 2023).

Target-dependent activation of the RuvC center can also trigger cleavage of non-target single-stranded DNA in trans, although this activity appears to depend strongly on the TnpB ortholog and experimental conditions. ISDra2 TnpB has been reported to cleave a fluorophore–quencher ssDNA reporter following activation by a cognate target, supporting the existence of Cas12-like collateral nuclease activity (Sasnauskas et al., 2023). More detailed characterization of the thermophilic SisTnpB1 ortholog from *Sulfolobus islandicus* demonstrated robust target-activated ssDNA trans-cleavage at approximately 75 °C, whereas activity was markedly reduced below 65 °C. Trans-cleavage was strongly impaired by mutations of the RuvC active-site residues D187 or E271 and was supported by either Mg^2+^ or Mn^2+^, with faster reporter cleavage observed in the presence of Mn^2+^. Mutations in the TAM or the TAM-proximal guide-matching region also strongly reduced activation (Xu et al., 2024). These findings establish collateral cleavage as a measurable biochemical property of at least some TnpB systems, although its prevalence across orthologs, physiological significance and relevance under genome-editing conditions remain insufficiently characterized.

Collectively, these findings support a model in which RuvC-mediated cleavage represents the terminal output of multiple upstream molecular checkpoints rather than an independently regulated catalytic event. TAM recognition licenses productive target engagement, progressive RNA–DNA pairing and DNA unwinding establish an appropriate substrate configuration, and conformational rearrangements around the RuvC lid are proposed to facilitate access to the catalytic center. DNA cleavage therefore emerges from coordination among target recognition, nucleic-acid remodeling and catalytic-site accessibility. This mechanistic coupling provides a direct basis for considering TnpB activity within the integrated protein–RNA constraint framework developed in Section 2.5.

### 2.5 Integrated protein–RNA coupled model of TnpB activity

The structural and biochemical evidence summarized above supports a coupled protein–RNA model in which productive TnpB activity emerges from interacting constraints arising from the nuclease architecture and its associated ωRNA/reRNA. The protein component provides the structural framework for TAM recognition, target DNA engagement, RNA–DNA heteroduplex stabilization, DNA unwinding and RuvC-mediated catalysis, whereas the RNA scaffold is required for stable ribonucleoprotein assembly, guide presentation and productive guide–target pairing. These functions are therefore mechanistically interdependent rather than separable modules, such that perturbation of either the protein or RNA component can influence multiple stages of target recognition and cleavage. The structural organization underlying TAM-dependent target engagement, RNA–DNA heteroduplex formation, and RuvC-mediated catalysis is summarized in Figure 1.

This coupling has direct consequences for genome-editor optimization. Protein substitutions that enhance target engagement or catalytic activation may also alter RNA interactions, TAM preference or sequence discrimination, whereas modifications of the ωRNA/reRNA scaffold can influence not only RNP formation but also heteroduplex propagation and access to cleavage-competent conformations. Similarly, the DNA-unwinding and RuvC-gating mechanisms described above indicate that increased target binding or guide complementarity alone may not translate directly into increased cleavage activity when another molecular checkpoint remains limiting. TnpB performance should therefore be viewed as an emergent property of compatibility among protein architecture, RNA configuration and target-dependent conformational transitions rather than as the sum of independently optimizable components.

This coupled-constraint framework provides the mechanistic basis for the engineering strategies discussed in Section 4. Natural ortholog diversity supplies alternative combinations of protein architecture, TAM preference and RNA scaffold organization, whereas protein engineering, TAM-recognition engineering and ωRNA/reRNA redesign directly modify molecular constraints within the TnpB RNP. Expression and delivery determine how efficiently these engineered molecular properties are realized in cellular environments, while specificity assessment determines whether gains in activity or target range are accompanied by acceptable fidelity. Accordingly, effective TnpB engineering requires coordinated optimization across these interacting layers, with engineered systems evaluated by overall editing performance rather than by improvement in any single molecular parameter.

## 3 Comparative landscape of RNA-Guided nucleases

### 3.1 Positioning TnpB within the expanding landscape of RNA-guided genome editors

Given the evolutionary and mechanistic relationship between TnpB and other RNA-guided nucleases, comparative analysis provides an evolutionary and mechanistic context for understanding the unique molecular constraints and engineering opportunities of TnpB. RNA-guided nucleases have diversified beyond canonical CRISPR-Cas systems, creating a broad design landscape defined by differences in molecular origin, protein architecture, guide RNA organization, target recognition mechanisms, cleavage architecture, specificity profiles, delivery compatibility, and engineering maturity (Makarova et al., 2020; Altae-Tran et al., 2021). Although Cas9 and Cas12 nucleases remain dominant genome editing platforms because of their established design principles and extensive derivative technologies, emerging compact nucleases provide alternative solutions for applications where delivery capacity and genetic payload size are limiting factors (Karvelis et al., 2021; Bigelyte et al., 2021).

Within this expanding landscape, TnpB occupies a unique position as a transposon-derived RNA-guided nuclease characterized by minimal protein architecture and a particularly strong dependence on coordinated protein–RNA interactions (Altae-Tran et al., 2021; Karvelis et al., 2021; Nakagawa et al., 2023). Unlike CRISPR-associated nucleases that evolved within adaptive immune systems, TnpB represents a mobile-element-derived editing scaffold in which target recognition depends on both TAM recognition by the protein component and functional organization of the ωRNA/reRNA scaffold (Sasnauskas et al., 2023; Marquart et al., 2024).

Therefore, TnpB should not be evaluated simply as a smaller version of Cas enzymes, but rather as a distinct nuclease architecture with its own engineering constraints and application opportunities. Accordingly, the following comparison considers TnpB not only in terms of molecular size, but also in relation to target recognition, catalytic organization, guide-RNA architecture, specificity, delivery constraints, and evolutionary origin.

### 3.2 Cas9: Established versatility with delivery limitations

Cas9 remains the benchmark RNA-guided nuclease because of its high activity, extensive engineering history, and broad application spectrum. Cas9-derived technologies have enabled not only gene disruption but also advanced editing modalities, including base editing, prime editing, transcriptional regulation, and epigenome modification (Jinek et al., 2012; Cong et al., 2013; Komor et al., 2016; Gaudelli et al., 2017; Anzalone et al., 2019). Beyond research applications, Cas9-based genome editing has recently achieved clinical validation. The approval of Casgevy (exagamglogene autotemcel, formerly CTX001), a CRISPR-Cas9-based therapeutic product for sickle cell disease and transfusion-dependent β-thalassemia, represents a milestone demonstrating the therapeutic potential of programmable genome editing (Frangoul et al., 2021; Leonard and Tisdale, 2024). These clinical advances highlight the maturity of Cas9 platforms, while also emphasizing the continued importance of developing smaller RNA-guided editors such as TnpB for applications where delivery capacity remains limiting.

However, the relatively large size of commonly used Cas9 orthologs represents a persistent limitation for delivery-constrained applications (Ran et al., 2015; Wang et al., 2024). Although smaller Cas9 variants and split systems have partially addressed this challenge (Truong et al., 2015; Ran et al., 2015), protein size remains an important factor when designing viral delivery systems or compact therapeutic vectors.

Mechanistically, Cas9 uses two distinct nuclease domains, with the HNH and RuvC active sites cleaving the target and non-target DNA strands, respectively, and target recognition is coupled to substantial conformational rearrangements during guide-RNA loading and R-loop propagation (Jinek et al., 2014; Nishimasu et al., 2014; Pacesa et al., 2022). By contrast, TnpB uses a single RuvC catalytic center to process both DNA strands, with cleavage coupled to TAM recognition, RNA–DNA heteroduplex formation, and conformational gating of the RuvC active site (Sasnauskas et al., 2023; Nakagawa et al., 2023). Compared with Cas9, TnpB provides a substantial reduction in nuclease size but currently lacks the same level of engineering maturity, including predictive guide design rules, established derivative platforms, and comprehensive specificity benchmarks (Karvelis et al., 2021; Thornton et al., 2026). Thus, Cas9 remains advantageous for applications requiring highly optimized and extensively validated editing, whereas TnpB offers potential advantages when molecular compactness is prioritized (Pickar-Oliver and Gersbach, 2019; Karvelis et al., 2021).

### 3.3 Cas12a: mature CRISPR platforms with diverse targeting properties

Cas12 nucleases represent another major class of RNA-guided editors with distinct molecular features from Cas9, including crRNA-based targeting and staggered DNA cleavage mediated by a single RuvC catalytic center (Zetsche et al., 2015). Several Cas12 family members have achieved substantial application maturity and have contributed both genome editing and molecular diagnostic technologies (Chen et al., 2018; Li et al., 2018).

Compared with TnpB, Cas12 systems benefit from more extensive biochemical characterization and established engineering strategies, including optimization of nuclease activity, target recognition, and guide RNA compatibility (Kleinstiver et al., 2019; Tsuchida et al., 2022). However, many Cas12 proteins remain relatively large, creating delivery challenges similar to Cas9 (Zetsche et al., 2015; Wu et al., 2021). Recent engineering efforts have further demonstrated that Cas12 optimization requires coordinated consideration of protein architecture and guide RNA design (Tsuchida et al., 2022).

The comparison between Cas12 and TnpB is particularly informative because structural studies suggest evolutionary relationships between these systems (Altae-Tran et al., 2021; Altae-Tran et al., 2023; Nakagawa et al., 2023). Both TnpB and Cas12 nucleases use a single RuvC catalytic center to cleave the target and non-target DNA strands, but their handling of the motif-distal DNA differs. In structurally characterized Cas12 enzymes, additional domains engage the distal RNA–DNA heteroduplex and re-hybridized DNA, facilitating further DNA unwinding and loading of the target strand into the RuvC active site. By contrast, ISDra2 TnpB lacks equivalent extensive contacts with the distal re-hybridized duplex and appears to rely more strongly on spontaneous DNA opening for target-strand capture (Nakagawa et al., 2023). Nevertheless, their engineering requirements differ substantially: Cas12 optimization also involves coordinated engineering of nuclease properties and guide RNA components within established CRISPR frameworks. Similarly, TnpB engineering requires optimization of both protein architecture and transposon-derived RNA scaffolds, with additional challenges arising from TAM recognition and ortholog-dependent protein–RNA compatibility (Kleinstiver et al., 2015; Walton et al., 2020; Thornton et al., 2026).

### 3.4 Cas12f and TnpB: compact nucleases with different engineering challenges

Cas12f represents one of the closest comparisons to TnpB because both systems occupy the compact nuclease space. Cas12f proteins are substantially smaller than canonical Cas9 and Cas12 nucleases and therefore offer advantages for delivery-limited applications (Karvelis et al., 2020; Bigelyte et al., 2021).

However, compactness alone does not determine practical utility. Native Cas12f systems often display limited activity and require substantial engineering to achieve efficient genome editing in eukaryotic contexts (Bigelyte et al., 2021). Similarly, TnpB performance depends strongly on optimization of protein architecture, TAM compatibility, and RNA scaffold function (Karvelis et al., 2021; Marquart et al., 2024).

The key distinction is their molecular origin and engineering logic. In structurally characterized systems, TnpB functions as a monomer associated with a single transposon-derived ωRNA/reRNA, whereas compact Cas12f nucleases such as UnCas12f1 function as dimers associated with a crRNA–tracrRNA guide complex. The second Cas12f protomer contributes to recognition of the PAM-distal region of an approximately 20-bp RNA–DNA heteroduplex, whereas ISDra2 TnpB primarily engages a shorter, approximately 12-bp heteroduplex within its monomeric architecture (Karvelis et al., 2021; Nakagawa et al., 2023). Consequently, Cas12f may be advantageous when compatibility with canonical CRISPR engineering principles is desired, while TnpB offers an alternative platform for exploring minimal RNA-guided nuclease architectures (Bigelyte et al., 2021; Karvelis et al., 2021).

### 3.5 IscB and Fanzor: Expanding the mobile-element-derived nuclease family

IscB and Fanzor systems provide important evolutionary context for understanding TnpB and demonstrate that programmable RNA-guided DNA targeting extends beyond bacterial CRISPR systems (Altae-Tran et al., 2021; Saito et al., 2023). These mobile-element-derived nucleases reveal diverse evolutionary solutions for RNA-guided DNA recognition (Altae-Tran et al., 2021).

Mechanistically, IscB is more Cas9-like than TnpB: it contains both RuvC and HNH nuclease domains, whereas TnpB relies on a single RuvC catalytic center (Kapitonov et al., 2015; Altae-Tran et al., 2021). Despite its compact protein architecture, IscB associates with a relatively large ωRNA scaffold that structurally compensates for the reduced proteinaceous recognition lobe and contributes directly to recognition of the guide–target heteroduplex (Kato et al., 2022). Fanzor, in contrast, represents a eukaryotic branch of the TnpB-related nuclease family and retains a RuvC-centered cleavage architecture, but characterized Fanzor proteins contain expanded REC and NUC elements and structurally distinct ωRNAs; for example, SpuFz1 accommodates an approximately 15-bp RNA–DNA heteroduplex and its ωRNA lacks the pseudoknot characteristic of ISDra2 TnpB (Saito et al., 2023).

Compared with these systems, TnpB currently represents one of the most compact and experimentally advanced mobile-element-derived nucleases (Karvelis et al., 2021; Nakagawa et al., 2023). However, its practical deployment remains dependent on improving target-site compatibility and editing efficiency, RNA design predictability, and editing fidelity (Marquart et al., 2024; Thornton et al., 2026).

### 3.6 Comparative positioning: Size, engineering burden, and application niche

The practical value of RNA-guided nucleases is determined not only by protein size but also by the balance between compactness, activity, engineering requirements, cleavage mechanism, specificity, delivery compatibility, evolutionary context, and application maturity (Jinek et al., 2014; Zetsche et al., 2015; Karvelis et al., 2020; Karvelis et al., 2021; Nakagawa et al., 2023).

A simplified comparison highlights distinct design niches (Table 1).

**TABLE 1:** Comparative positioning of TnpB among RNA-guided nuclease systems.

System	Approx. Size	Guide/motif	Cleavage architecture	Editing and specificity maturity	Delivery profile	Evolutionary position/main limitation
Cas9	∼1000–1600 aa	sgRNA/PAM	DSB; HNH and RuvC domains cleave opposite DNA strands	High; extensive activity and off-target benchmarks	Large size can constrain single-vector delivery; compact orthologs and split systems available	Type II CRISPR effector; mature platform but relatively large
Cas12a	∼1000–1300 aa	crRNA/PAM	Staggered DSB with 5′ overhangs; single RuvC catalytic center	High–moderate; extensively characterized for major orthologs	Delivery remains size-limited for many Cas12 proteins	Type V CRISPR effector; evolutionarily related to TnpB but generally larger
Cas12f	∼400–700 aa	crRNA-based/PAM	DSB; single RuvC-centered cleavage architecture; representative systems function as compact dimers	Emerging; specificity less extensively benchmarked than Cas9/Cas12a	Compact architecture favors size-constrained delivery	Compact Type V CRISPR effector; native activity often requires substantial engineering
TnpB	∼350–400 aa	ωRNA/reRNA/TAM	Staggered DSB with 5′ overhangs; single RuvC catalytic center processes both DNA strands	Emerging; mismatch and genome-wide specificity data are increasing but not yet standardized	Highly compatible with compact constructs; single-AAV delivery demonstrated	Likely evolutionary precursor of Cas12; TAM and RNA-scaffold constraints remain major limitations
IscB	variable, often compact-intermediate	ωRNA/TAM	DSB; HNH and RuvC nuclease domains	Early; genome-editing and specificity validation remain limited	Compact protein architecture, but delivery performance is less established	OMEGA-family nuclease and likely evolutionary precursor of Cas9; limited editing maturity
Fanzor	variable, compact-intermediate	ωRNA/motif-dependent	DSB; RuvC-centered cleavage architecture	Early; comparatively limited editing and off-target evidence	Delivery advantages remain insufficiently characterized	Eukaryotic TnpB-related nuclease; activity and engineering evidence remain limited

Comparison of representative CRISPR-associated and mobile-element-derived RNA-guided nucleases according to protein size, guide and target-motif architecture, DNA cleavage mechanism, editing and specificity maturity, delivery profile, evolutionary position, and major remaining limitations. Representative references for each system are as follows: Cas9 (Jinek et al., 2014; Nishimasu et al., 2014; Pacesa et al., 2022); Cas12a (Zetsche et al., 2015; Stella et al., 2018); Cas12f (Karvelis et al., 2020; Bigelyte et al., 2021; Xu et al., 2021; Wu et al., 2021); TnpB (Karvelis et al., 2021; Sasnauskas et al., 2023; Nakagawa et al., 2023; Marquart et al., 2024); IscB (Kato et al., 2022); and Fanzor (Saito et al., 2023) Abbreviations: sgRNA, single-guide RNA; crRNA, CRISPR RNA; PAM, protospacer-adjacent motif; TAM, transposon-associated motif; DSB, double-strand break; AAV, adeno-associated virus.

Rather than competing directly with mature CRISPR systems, TnpB occupies a distinct design space where minimal architecture and delivery flexibility are primary advantages (Karvelis et al., 2021; Marquart et al., 2024). Future engineering efforts will determine whether these advantages can be translated into reliable and scalable genome editing applications.

### 3.7 Future position of TnpB in genome editing biotechnology

The emerging landscape of RNA-guided nucleases suggests that no single editor will dominate all applications. Large but highly optimized nucleases such as Cas9 and Cas12 will likely remain central for many genome engineering applications, whereas compact editors such as Cas12f and TnpB may provide specialized solutions for delivery-restricted contexts.

Recent advances in engineered TnpB variants, optimized ωRNA/reRNA scaffolds, and expanded biological applications indicate that TnpB is transitioning from a naturally occurring transposon-associated nuclease toward an engineered biotechnology platform (Marquart et al., 2024; Li et al., 2026; Nagalakshmi et al., 2026). Its future development will depend on whether improvements in activity and target range can be integrated with predictable protein–RNA compatibility, editing specificity, and delivery performance.

## 4 Engineering strategies for TnpB optimization

Native TnpB nucleases provide a compact RNA-guided DNA cleavage platform, but their direct application as genome editing tools remains limited by heterogeneous activity, restrictive TAM requirements, incomplete guide RNA design rules, host-dependent expression, and insufficiently characterized specificity profiles (Karvelis et al., 2021; Sasnauskas et al., 2023). These limitations indicate that TnpB performance is not determined by a single molecular parameter, but instead reflects the coordinated behavior of protein architecture, TAM recognition, guide RNA scaffold integrity, intracellular expression, delivery format, and DNA repair context.

Current engineering efforts have focused mainly on identifying active orthologs and improving the performance of existing TnpB systems. More recent studies have begun to address TAM flexibility, RNA scaffold design, and delivery-related constraints (Altae-Tran et al., 2023; Thornton et al., 2026). These strategies address different bottlenecks in TnpB development. Ortholog screening provides access to naturally diverse enzymes, whereas protein and RNA engineering are required to optimize activity in heterologous systems (Karvelis et al., 2021; Marquart et al., 2024; Thornton et al., 2026). Together, these strategies define the current optimization landscape for transforming TnpB from a naturally occurring transposon-associated nuclease into a programmable genome editing platform.

### 4.1 Natural diversity and ortholog selection as an engineering resource

Natural TnpB diversity provides a broad sequence and functional space from which genome-editing candidates can be selected before further protein or RNA engineering (Xiang et al., 2024; Feng et al., 2024; Ren et al., 2024). Systematic screening of IS605-associated TnpB systems has revealed substantial divergence in protein sequence, TAM preference, reRNA architecture and cellular activity (Xiang et al., 2024; Feng et al., 2024). Xiang et al. screened TnpBs from 64 annotated IS605 elements and identified 25 systems with detectable activity in *Escherichia coli*, three of which were also active in human cells. Subsequent *de novo* annotation identified 14 additional candidates, among which ISAam1 TnpB (369 aa) and ISYmu1 TnpB (382 aa) showed robust editing across multiple endogenous loci in human cells and achieved overall editing performance comparable to the substantially larger SaCas9 in the assays performed (Xiang et al., 2024). These results demonstrate that ortholog mining can reveal compact editors with substantially different functional properties even before deliberate sequence engineering.

Differences among orthologs extend beyond protein size to target-motif compatibility and host-dependent performance. ISDra2 TnpB, the most extensively characterized structural model, is a 408-aa nuclease with a strong preference for the 5′-TTGAT-3′ TAM. ISAam1 and ISYmu1 provide alternative compact scaffolds with distinct targeting characteristics; for example, plant genome-editing experiments have used a 5′-TTTAA-3′ TAM for ISAam1, whereas ISYmu1 and ISDra2 were targeted to 5′-TTGAT-3′ sites. Notably, the three systems did not perform equivalently in the same plant context: ISDra2 and ISYmu1 produced detectable editing in rice, whereas ISAam1 showed no detectable editing under the conditions tested (Li Q. et al., 2024). Thus, favorable activity in one host cannot be assumed to predict equivalent performance after transfer to another biological system (Li Q. et al., 2024).

Natural ortholog diversity can also expand targeting scope without requiring protein mutagenesis. SisTnpB7 from the thermophilic archaeon *Sulfolobus islandicus* recognizes the cognate 5′-TTTAA-3′ TAM but can also support genome editing at a broad range of weaker TAM variants. For example, a 5′-GTTAC-3′ TAM exhibiting only 11.5% relative *in vitro* cleavage activity nevertheless supported >80% genome editing, whereas targets flanked by CTTGA and ATTGA supported targeted gene deletion efficiencies of approximately 91% and 82%, respectively (Feng et al., 2024). The same system also enabled homology-directed single-nucleotide editing and remained functional after transfer to mesophilic bacterial hosts, although editing outcomes depended strongly on TAM strength, expression level and host context. These observations illustrate that ortholog suitability cannot be ranked solely by maximal biochemical cleavage activity; TAM flexibility, cellular context and the intended editing outcome can be equally important selection criteria.

The natural design space of TnpB has been further expanded by characterization of IS607-associated systems, which differ substantially from the more extensively studied IS200/IS605 TnpBs (Ren et al., 2024). IS607 TnpBs associated with RAGATH-18-derived reRNAs have an average protein length of approximately 390 aa, with characterized members ranging from 330 to 433 aa. ISFba1 TnpB from Firmicutes bacterium AM43-11BH is a 387-aa representative that recognizes a G-rich 5′-AGGAG-3′ TAM. More broadly, analysis of IS607 TnpBs from 22 bacterial strains identified 5′-AGGAG-3′ and 5′-GAGGG-3′ as the predominant TAM classes. In HEK293F cells, ISFba1 TnpB produced indel frequencies of 42.5% at EMX1-T1, 17.0% at EMX1-T2 and 17.9% at VEGFA-T1, demonstrating that naturally occurring TnpB systems with TAM preferences distinct from the AT-rich motifs of ISDra2 can support substantial mammalian genome-editing activity (Ren et al., 2024).

The broader TnpB-related sequence space also includes naturally exapted, nuclease-deficient homologues. TnpB-like nuclease-dead repressors (TldRs) use naturally occurring guide RNAs for sequence-specific DNA binding and transcriptional repression rather than DNA cleavage, illustrating that evolutionary diversification of TnpB-like systems can generate molecular functions beyond endonuclease activity (Wiegand et al., 2024). Taken together, currently characterized orthologs do not identify a single universally superior TnpB scaffold (Xiang et al., 2024; Feng et al., 2024; Ren et al., 2024). Rather, they provide distinct starting points for engineering: ISDra2 offers the best-developed structural and mechanistic framework; ISAam1 and ISYmu1 provide compact alternatives with strong mammalian-cell activity; SisTnpB7 illustrates the potential value of naturally flexible TAM recognition for microbial editing; and IS607 systems such as ISFba1 extend the family toward G-rich TAMs and structurally distinct protein–RNA architectures (Xiang et al., 2024; Feng et al., 2024; Ren et al., 2024). Ortholog selection should therefore be treated as a multi-parameter design decision integrating protein size, TAM compatibility, RNA architecture, editing activity and host context before subsequent protein or RNA optimization is undertaken.

### 4.2 Protein engineering of activity and target recognition

Protein engineering provides a major route for improving TnpB performance by modulating nuclease activity, protein stability, RNA interaction, target DNA engagement, and TAM-dependent recognition (Thornton et al., 2026; Marquart et al., 2024; Skopintsev et al., 2026). Because the compact TnpB architecture integrates RNA- and DNA-interacting regions with a RuvC catalytic core, substitutions at individual positions can influence multiple steps of the reaction pathway, including ribonucleoprotein assembly, RNA–DNA heteroduplex stabilization, target recognition, catalytic activation, and DNA cleavage (Sasnauskas et al., 2023; Nakagawa et al., 2023). Accordingly, engineering interventions at one molecular interface may propagate across several functional steps, supporting the view that TnpB optimization should be considered as a coupled rather than strictly modular process (Sasnauskas et al., 2023; Thornton et al., 2026). Structural studies of ISDra2 TnpB, for example, identify a set of residues in the REC and WED domains that directly participate in TAM recognition and strongly affect cleavage activity when mutated (Nakagawa et al., 2023). More recent studies have demonstrated that the TnpB protein sequence itself contains substantial latent engineering potential. Thornton et al. performed deep mutational scanning across the ISDra2 TnpB ribonucleoprotein and subsequently combined activity-enhancing substitutions to generate highly active variants. Two selected variants, TnpB-KYLI (N4Y/R110K/V192L/L222I) and TnpB-VGIRL (L172G/V192L/L222I/P282V/I304R), increased editing across human and plant systems. At the *NbNDR1* locus in *Nicotiana benthamiana*, TnpB-KYLI and TnpB-VGIRL reached approximately 55% and 49% indel frequencies, respectively, compared with <1% for WT ISDra2 TnpB, corresponding to improvements exceeding 50-fold at this target (Thornton et al., 2026). The mutational landscape further revealed activity-enhancing substitutions in regions surrounding nucleic-acid-interacting surfaces, illustrating how systematic sequence–function mapping can uncover engineering opportunities that are difficult to predict from static structures alone.

Complementary work has shown that iterative selection directly in mammalian cells can also identify highly active TnpB variants (Gorbenko et al., 2026). Gorbenko et al. used repeated mutagenesis and selection for improved editing to generate TnpBSuper, whose final configuration contains nine protein-coding mutations together with mammalian codon optimization and an optimized Trim2 ωRNA. The evolved system achieved improvements of up to approximately 11-fold over the parental TnpB in the assays used, and enhanced activity was maintained across endogenous mammalian targets. These results emphasize that selection environment can influence the mutations recovered: mammalian-cell evolution and yeast-based mutational scanning identified some convergent substitutions, such as changes involving V192 and L172, but also produced substantially different mutational solutions (Gorbenko et al., 2026; Thornton et al., 2026). More recently, structure- and evolution-guided protein design has expanded TnpB engineering beyond naturally occurring sequence variation. Skopintsev et al. combined structure-guided inverse folding with evolution-informed residue constraints to generate synthetic TnpB variants (SynTnpBs), several of which retained or exceeded wild-type activity across bacterial, plant, and human cells. Cryo-EM analysis of a divergent active SynTnpB further identified stabilizing contacts at RNA–DNA interfaces, demonstrating that computational protein design can access functional TnpB sequence space beyond naturally evolved variants (Skopintsev et al., 2026).

Protein engineering can also directly alter the target-recognition landscape of TnpB. Structural analysis of ISDra2 TnpB identified K76 and Q80 in the REC domain as key contacts with the TAM, providing a molecular basis for structure-guided reprogramming (Sasnauskas et al., 2023; Nakagawa et al., 2023). Marquart et al. therefore performed saturation mutagenesis at positions 76 and 80. Although Q80 substitutions did not substantially improve recognition of the alternative TAMs tested, six K76 variants—K76A, K76C, K76G, K76R, K76S, and K76T—showed more than fivefold higher activity than TnpBmax against at least one alternative TAM in biochemical profiling. In HEK293T cells, K76A, K76C, and K76S supported >17% editing at a non-canonical 5′-TTTAT-3′ TAM, with TnpBmax-K76A reaching 21.2%. Broader profiling showed that K76 engineering shifted TAM preference away from the canonical 5’ -TTGAT-3′ motif and enabled recognition of a broader 5’ -TYKAT-3′ motif class, substantially expanding the potential targeting range of ISDra2 TnpB (Marquart et al., 2024).

Importantly, increased nuclease activity or expanded target recognition should not be assumed to produce an unavoidable loss of specificity. TnpB-KYLI and TnpB-VGIRL showed off-target editing at predicted sites in *N. benthamiana* that was comparable to or lower than WT ISDra2 TnpB under the conditions tested, whereas mammalian-cell-evolved TnpBSuper did not generate additional off-target sites in the DISCOVER-Seq analyses performed in that study (Thornton et al., 2026; Gorbenko et al., 2026). Nevertheless, these datasets remain limited in ortholog diversity, target number, host context, and profiling methodology. Protein engineering of TnpB should therefore be evaluated jointly across activity, target range, and specificity, with quantitative on-target measurements, TAM profiling, mismatch-tolerance assays, and unbiased genome-wide off-target analyses used to determine whether apparent activity gains represent genuine improvements in overall editing performance (Gorbenko et al., 2026; Thornton et al., 2026).

### 4.3 Engineering the ωRNA/reRNA scaffold as a functional component of TnpB editing

The ωRNA/reRNA scaffold represents an essential molecular component of the TnpB editing system rather than a passive guide RNA (Karvelis et al., 2021; Nakagawa et al., 2023; Li Z. et al., 2024). Through conserved structural elements, the scaffold enables TnpB ribonucleoprotein assembly, guide positioning, and productive target recognition (Karvelis et al., 2021; Sasnauskas et al., 2023). Conserved RNA elements in ISDra2 include a pseudoknot, stem one and an associated triple-helix structure, together with additional stem elements that organize interactions with the WED and RuvC domains and position the guide segment for target DNA pairing (Nakagawa et al., 2023; Sasnauskas et al., 2023). However, not all regions of the scaffold are equally constrained; for example, stem 3b of the ISDra2 ωRNA is unresolved in the ternary structure, consistent with greater structural flexibility. RNA architecture also varies across TnpB clades: IS607-associated TnpBs use structurally distinct RAGATH-18-derived reRNAs, and the ISFba1 reRNA contains three stem loops and two pseudoknots, with disruption of PK1 abolishing DNA cleavage. As a result, RNA scaffold integrity strongly influences editing performance.

RNA engineering strategies include optimization of guide length, stabilization of conserved secondary structures, modification of scaffold boundaries, and improvement of RNA folding stability. These approaches aim not only to enhance guide availability but also to improve RNP assembly, conformational activation, and coordination between RNA-guided recognition and TnpB catalytic function. Structure-guided truncation of the ISDra2 ωRNA provided direct evidence that flexible scaffold regions can be removed without compromising activity. Deletion of the disordered 5′ region from nucleotides −231 to −117 generated the Trim1 scaffold, which retained editing activity similar to or higher than that of the full-length ωRNA. Further deletion of the structurally unresolved stem 3b region (−70 to −49), with the remaining termini connected by a GAAA linker, generated Trim2 and further enhanced genome-editing activity at several endogenous human loci (Nakagawa et al., 2023). A complementary stepwise engineering strategy divided the 231-nt ISDra2 ωRNA into six predicted segments (S1–S6). Individual deletion analysis showed that S4 and S6 were essential for TnpB activity, whereas S1, S2, S3 and S5 were more tolerant to removal. Simultaneous deletion of S1–S3 generated ωRNA-v1 with increased activity, and subsequent partial replacement within the SL3 region with a 5′-GAAA-3′ loop contributed to the optimized ωRNA-v2 (ωRNA*). Compared with the cognate ωRNA, ωRNA* significantly increased editing at 10 of 14 endogenous loci tested in HEK293T cells and produced an approximately twofold increase in editing efficiency across four targets in mouse N2a cells (Li Z. et al., 2024). In addition, guide sequence composition and positional mismatch tolerance are likely to influence both efficiency and specificity, making guide design a critical determinant of TnpB performance.

Compared with the extensively characterized guide RNA design principles in Cas9 and Cas12 systems, the design principles governing TnpB ωRNA/reRNA scaffolds remain relatively immature (Marquart et al., 2024; Thornton et al., 2026). Because the ωRNA/reRNA scaffold simultaneously functions as a structural organizer and a targeting element, optimization requires understanding both RNA folding constraints and guide-mediated target recognition. Nevertheless, recent high-throughput studies have begun to establish quantitative design rules. Marquart et al. profiled TnpBmax editing efficiencies across 10,211 target sites and used these data to develop the TnpB editing efficiency predictor (TEEP), which predicts ISDra2 ωRNA activity with a reported correlation exceeding 0.8 (Marquart et al., 2024). Complementary deep mutational scanning of a 116-nt ISDra2 reRNA scaffold across 576 variants identified activating substitutions and deletions concentrated around the stem two hinge region, particularly near the unpaired nucleotides rA−40–rU−43. Hinge-region deletions enhanced EGFP knockout relative to the WT reRNA in HEK293T cells, whereas the Trim2 scaffold, despite strong enrichment in yeast selection, showed reduced activity in the mammalian assay, indicating that beneficial RNA modifications can be assay- and host-context dependent (Thornton et al., 2026). Importantly, RNA and protein improvements were not necessarily additive: pairing the highly active eTnpBd protein variant with either of two hinge-deletion reRNAs reduced EGFP editing to approximately 15%–24%, compared with 44.5% when eTnpBd was paired with the WT reRNA (Thornton et al., 2026). This provides direct evidence that TnpB performance depends on compatibility between engineered protein and RNA components rather than on independent optimization of either component alone (Thornton et al., 2026). Despite these advances, transferable rules for guide length, sequence composition, secondary structure, scaffold architecture, and protein–RNA compatibility remain incompletely defined across different TnpB orthologs and host systems (Marquart et al., 2024; Thornton et al., 2026). Future work should integrate structure-guided RNA design, high-throughput screening, RNA structure profiling, and computational prediction models to establish transferable design principles for ωRNA/reRNA scaffolds across TnpB orthologs (Marquart et al., 2024; Thornton et al., 2026). Beyond rational engineering of guide RNAs, evolutionary diversification provides additional insights into how TnpB-associated RNA architectures can be reorganized. In addition to experimental scaffold engineering, natural evolutionary diversification provides complementary evidence for the structural plasticity of TnpB-associated guide RNAs. Recent evolutionary analysis identified TranC intermediates derived from IS605- and IS607-associated TnpB lineages, in which the ancestral reRNA architecture is functionally split into tracrRNA and crRNA, illustrating how TnpB-associated RNA architectures can be reorganized during the evolutionary transition toward type V CRISPR-Cas effectors (Jin et al., 2025).

### 4.4 Expression, construct, and delivery optimization

The compact size of TnpB, typically around 350–400 amino acids, provides a major advantage for delivery-constrained genome editing applications (Wang et al., 2024; Marquart et al., 2024; Thornton et al., 2026). Because of its compact size, TnpB is particularly compatible with size-limited delivery systems, including adeno-associated virus (AAV) vectors and compact all-in-one expression constructs (Li Z. et al., 2024; Marquart et al., 2024). This delivery advantage has already been demonstrated *in vivo*. An optimized ISDra2 TnpB–ωRNA system was packaged into a single AAV vector and used to edit the *Hpd* locus in a *Fah*-deficient mouse model of hereditary tyrosinaemia; liver indel frequencies reached approximately 20% with the cognate ωRNA and 60% with the optimized ωRNA 1 month after treatment, accompanied by phenotypic rescue (Li Z. et al., 2024). More recently, AAV delivery of TnpBmax combined with computationally selected ωRNAs achieved editing efficiencies of up to 75.3% in mouse liver and 65.9% in mouse brain, further demonstrating that the compact TnpB architecture can translate into high-efficiency *in vivo* delivery rather than merely providing a theoretical packaging advantage (Marquart et al., 2024). This feature provides an important motivation for developing TnpB-based editors.

However, small protein size alone does not guarantee efficient editing. Cellular performance depends on codon optimization, promoter strength, RNA expression format, nuclear localization signal design, protein stability, and the stoichiometry between TnpB and its guide RNA. A representative example is TnpBmax, an optimized ISDra2 TnpB system developed for mammalian genome editing through construct and expression optimization rather than amino-acid substitutions in the nuclease core. Mammalian codon optimization of ISDra2 TnpB increased editing activity by approximately 3.3-fold across a 94-site target-matched library, whereas addition of a GS linker and a C-terminal bipartite nuclear localization signal further increased activity by approximately 1.3-fold, resulting in an average 4.4-fold improvement for the optimized TnpBmax configuration relative to the original construct (Marquart et al., 2024). Western blot analysis indicated that the increased activity of TnpBmax was associated primarily with higher cellular protein abundance rather than increased nuclear localization, emphasizing protein expression and stability as important determinants of TnpB performance. In eukaryotic systems, additional constraints such as chromatin accessibility, subcellular trafficking, and host DNA repair pathways further affect editing efficiency (Hsu et al., 2013).

Different biological systems require different delivery strategies. In microbial systems, plasmid-based delivery remains relatively straightforward and experimentally tractable. For example, SisTnpB7 has been deployed using plasmid-based expression and repair-template constructs in both its native archaeal host and heterologous bacterial systems, supporting homology-directed genome editing without requiring specialized delivery platforms (Feng et al., 2024). In plant systems, TnpB delivery has been demonstrated through protoplast transfection and *Agrobacterium*-mediated transformation. In rice, binary-vector delivery by *Agrobacterium* generated edited regenerated plants, including monoallelic editing in 38% of T_0_ plants at the *HMBPP* locus (Karmakar et al., 2024). More recently, transient *Agrobacterium* infiltration was used to deliver engineered TnpB variants into pepper leaves, where TnpB-VGIRL produced editing efficiencies of up to 10% at *CaAGO2* compared with <1% for WT TnpB (Thornton et al., 2026). In mammalian systems, plasmid transfection remains widely used for cellular screening, whereas delivery beyond cultured cells has been demonstrated by co-injection of TnpB mRNA and ωRNA into mouse embryos and by single-AAV delivery to somatic tissues (Li Z. et al., 2024; Marquart et al., 2024). Future optimization should therefore consider delivery as an integral component of TnpB system engineering (Li Z. et al., 2024; Marquart et al., 2024).

### 4.5 Specificity control and off-target evaluation

Specificity is a critical requirement for any genome editing system, particularly for applications in mammalian cells, plants, and therapeutic contexts (Fu et al., 2013; Hsu et al., 2013; Tsai et al., 2015; Kim et al., 2015). For TnpB, available structural and functional evidence indicates that specificity is shaped by TAM recognition and position-dependent guide RNA–DNA complementarity, in addition to the intracellular abundance and duration of exposure of the active nuclease complex. In ISDra2 TnpB, double mismatches within the TAM-proximal positions 1–12 abolished detectable *in vitro* target cleavage, whereas double mismatches at the TAM-distal positions 13–16 reduced but did not eliminate cleavage (Nakagawa et al., 2023). Consistent with this positional asymmetry, genome-wide analysis in human cells identified multiple off-target sites containing mismatches preferentially within the TAM-distal region, indicating that approximately the first 12 bp of the RNA–DNA heteroduplex constitute a major specificity-determining region. Thus, although TnpB specificity remains less extensively characterized compared with established Cas9 and Cas12 systems, its mismatch-sensitivity landscape is beginning to be experimentally defined rather than remaining purely predictive.

Existing CRISPR off-target profiling methods provide a useful methodological foundation for evaluating TnpB fidelity, and both targeted and genome-wide approaches have now been applied to TnpB systems, although standardized TnpB-specific benchmarking remains limited (Wang et al., 2024; Marquart et al., 2024; Thornton et al., 2026). For example, GUIDE-seq analysis of TnpBmax paired with four different ωRNAs in HEK293T cells detected off-target cleavage for only two of the four guides, and the identified off-target sites lacked mismatches at the TAM-proximal end of the guide, consistent with the strong mismatch sensitivity of the approximately 12-bp seed region (Marquart et al., 2024). *In vivo* analyses have likewise provided initial evidence of high fidelity: after AAV-mediated ISDra2 TnpB editing of the *Pcsk9* and *Rosa26* loci in mouse liver, targeted deep sequencing of five predicted off-target sites for each locus and whole-genome sequencing of hepatocytes detected no evident off-target editing under the conditions tested (Wang et al., 2024). Because TnpB uses distinct guide RNA scaffolds and TAM-dependent recognition, off-target behavior may not fully mirror that of canonical CRISPR-Cas nucleases. Importantly, enhanced on-target activity can alter this balance. When six predicted genomic off-target sites containing four to six guide mismatches were examined for engineered ISDra2 TnpB variants, all tested variants showed increased off-target editing relative to WT ISDra2 or ISYmu1 at at least two sites; the highest observed off-target indel frequency reached approximately 6% for eTnpBc, whereas eTnpBe remained below 2% at all tested off-target sites (Thornton et al., 2026). These results indicate that TnpB specificity is already experimentally measurable but remains strongly dependent on guide sequence, mismatch position and the activity of the engineered protein variant.

Computational guide design, mismatch sensitivity profiling, transient expression formats, and activity–specificity balancing during protein and RNA engineering may help improve specificity. However, these strategies should be benchmarked using both targeted validation and unbiased genome-wide detection methods. Although current studies generally indicate favorable specificity for several TnpB systems, the available evidence remains based on a limited number of orthologs, guides, target loci, and assay formats, making direct comparison across studies difficult. For future applications, especially in animals and potential therapeutic settings, rigorous specificity assessment and standardized benchmarking across WT and engineered TnpB variants will be essential for determining whether TnpB can be safely deployed beyond proof-of-concept experiments.

### 4.6 Engineering framework summary

TnpB optimization should be evaluated as a multi-parameter engineering problem. Protein mutations, TAM reprogramming, ωRNA/reRNA scaffold redesign, expression tuning, and delivery configuration all act on the same compact ribonucleoprotein complex and can therefore generate coupled trade-offs (Marquart et al., 2024; Thornton et al., 2026). TAM relaxation may expand target range but reduce motif discrimination, whereas activity-enhancing mutations or prolonged expression may increase off-target exposure. Likewise, RNA-scaffold modifications that improve activity in one protein or host context may not remain beneficial when combined with independently optimized TnpB variants, further emphasizing the interdependence of protein and RNA engineering. These trade-offs argue for parallel benchmarking of activity, TAM compatibility, ωRNA/reRNA scaffold robustness, host transferability, delivery feasibility, and genome-wide specificity. Accordingly, TnpB engineering is best approached as coordinated optimization across natural scaffold selection, protein and TAM engineering, RNA-scaffold design, expression and delivery, and specificity control, rather than as sequential optimization of independent modules. The major constraint layers and corresponding engineering strategies for optimizing TnpB activity are summarized in Table 2.

**TABLE 2:** Engineering strategies for optimizing coupled protein–RNA constraints in TnpB genome editing.

Constraint layer	Engineering strategy	Molecular target	Expected benefit	Remaining challenge	Representative examples/references
Natural diversity and ortholog selection	Ortholog mining and functional screening	Protein scaffold, cognate TAM, and ωRNA/reRNA architecture	Identifies compact editors with alternative TAM preferences and host-adapted activity	Performance remains strongly ortholog-, target-, and host-dependent	ISDra2, ISAam1, ISYmu1, SisTnpB7, ISFba1 (Xiang et al., 2024; Feng et al., 2024; Ren et al., 2024)
TnpB protein architecture	Rational design, mutational scanning, combinatorial engineering, and directed evolution	RNA-binding regions, TAM recognition interface, RuvC catalytic domain	Improves RNP assembly, target recognition, catalytic activity, and protein stability	Beneficial mutations may affect specificity or activity balance	TnpB-KYLI, TnpB-VGIRL, TnpBSuper, SynTnpBs (Thornton et al., 2026; Gorbenko et al., 2026; Skopintsev et al., 2026)
Protein-mediated target recognition	TAM compatibility engineering	TAM-interacting residues and DNA-recognition interface	Expands targetable sequence space and enables recognition of alternative TAMs	Relaxed TAM recognition may reduce sequence discrimination	TnpBmax-K76A/K76C/K76S (Sasnauskas et al., 2023; Marquart et al., 2024)
ωRNA/reRNA scaffold	Scaffold engineering, guide optimization, RNA structure stabilization	Conserved scaffold core, flexible stem regions, and guide architecture	Enhances RNP assembly, guide presentation, and editing efficiency	RNA design rules remain ortholog-dependent	Trim1, Trim2, RNA-v1/RNA-v2, stem-2 hinge variants (Nakagawa et al., 2023; Li et al., 2024b; Thornton et al., 2026)
Protein–RNA compatibility	Co-optimization of TnpB variants and RNA scaffolds	Protein–RNA interaction interface	Balances activity gains across engineered protein and RNA components	Beneficial protein and RNA modifications may not be additive	Engineered TnpB–reRNA combinations; TnpBSuper + Trim2 (Thornton et al., 2026; Gorbenko et al., 2026)
Expression and delivery context	Codon optimization, promoter selection, NLS engineering, vector design	Cellular abundance and localization	Improves activity in diverse biological systems	Excessive expression may increase off-target effects	TnpBmax; single-AAV TnpB systems (Li et al., 2024b; Marquart et al., 2024; Wang et al., 2024)
Specificity control	Genome-wide profiling, high-fidelity engineering, computational design	TAM dependence, mismatch tolerance, exposure duration	Improves editing fidelity and safety	Standardized TnpB-specific benchmarks across orthologs and engineered variants remain limited	TnpBmax, eTnpBc, eTnpBe (Wang et al., 2024; Marquart et al., 2024; Thornton et al., 2026)

Summary of major engineering layers, representative strategies, expected benefits, and current limitations in TnpB optimization.

These trade-offs motivate a qualitative activity landscape in which TAM compatibility and ωRNA/reRNA scaffold integrity define the permissive editing regime (Figure 2).

**FIGURE 2:**
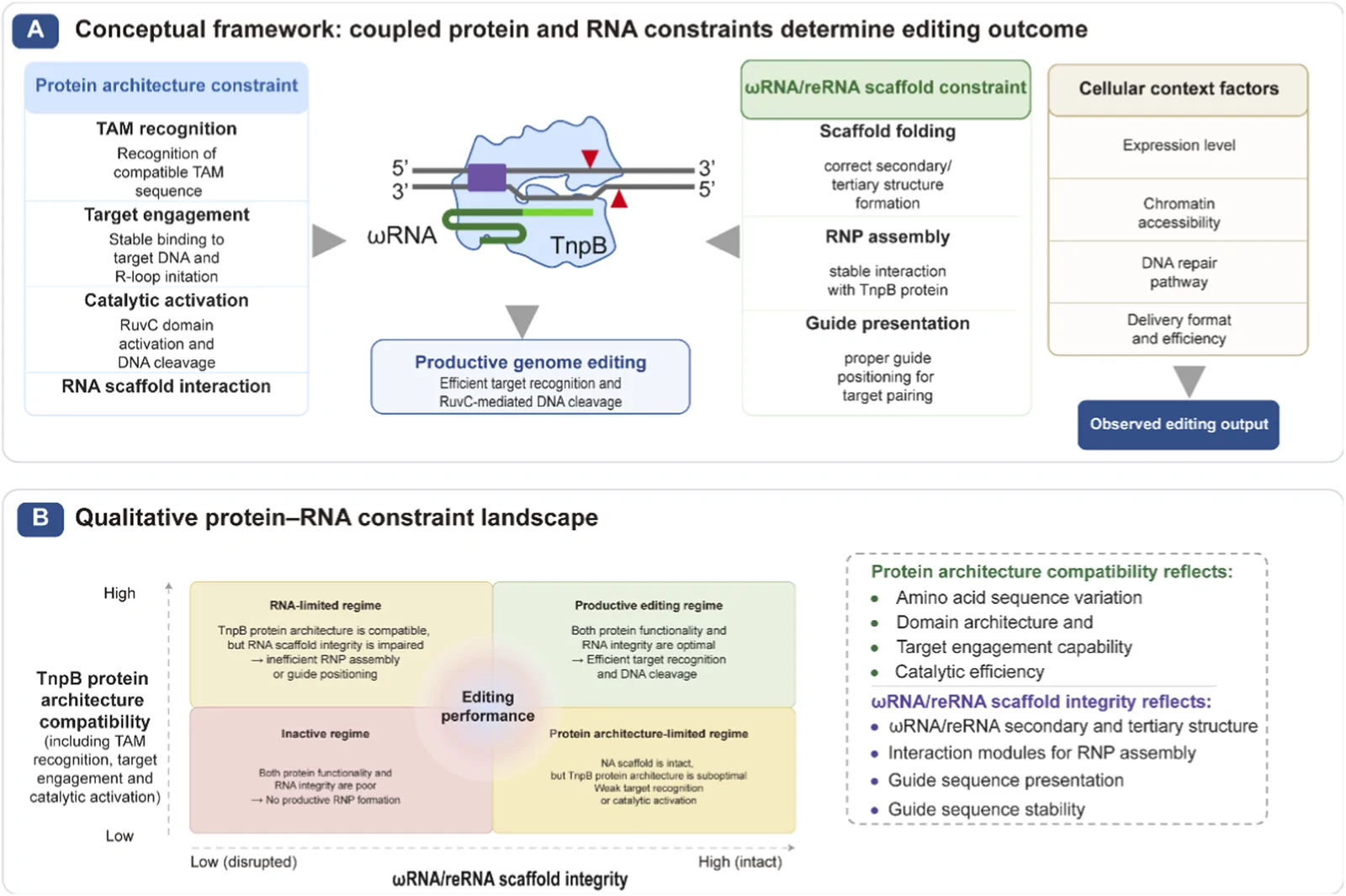
Coupled protein–RNA constraint landscape governing TnpB-mediated genome editing performance. TnpB editing performance is determined by the coordinated compatibility between two molecular constraints: TnpB protein architecture and ωRNA/reRNA scaffold integrity. **(A)** Productive editing requires functional TnpB architecture, including TAM-dependent target recognition, DNA engagement, RNA interaction, and RuvC-mediated cleavage, together with an intact ωRNA/reRNA scaffold supporting RNP assembly and guide presentation. Cellular factors further modulate the observed editing outcome. **(B)** The qualitative protein–RNA constraint landscape illustrates how disruption of either constraint limits productive editing, whereas simultaneous optimization enables efficient genome modification. This conceptual framework highlights engineering principles for improving TnpB editors rather than providing a quantitative predictive model.

## 5 Applications of TnpB-Mediated genome editing

### 5.1 Application maturity and biotechnology potential of TnpB platforms

TnpB-mediated genome editing has rapidly expanded from mechanistic characterization toward applications in diverse biological systems, including microorganisms, plants, and mammalian cells (Karvelis et al., 2021; Li Q. et al., 2024; Li Z. et al., 2024; Thornton et al., 2026). However, the practical maturity of TnpB platforms differs substantially among biological contexts. Unlike established CRISPR-Cas editors, whose applications are supported by extensive optimization pipelines and standardized design principles, TnpB remains an emerging technology whose performance depends strongly on nuclease identity, TAM compatibility, RNA scaffold configuration, delivery strategy, and host cellular environment.

A useful framework for evaluating TnpB applications is therefore not simply the presence of successful editing events, but the overall engineering readiness of the platform. Key parameters include editing efficiency, reproducibility across genomic loci, scalability of delivery, predictability of guide design, specificity characterization, and compatibility with downstream biotechnology applications.

Currently, bacterial systems represent the most experimentally mature context for TnpB development because of their tractable genetics, efficient transformation methods, and suitability for rapid screening. Plant systems represent an emerging application area where the compact size of TnpB provides potential advantages for transient and viral delivery approaches. Mammalian and *in vivo* applications remain comparatively less mature but highlight the potential of TnpB for delivery-constrained genome engineering, particularly in contexts where vector capacity limits the use of larger editors.

Together, these application landscapes suggest that TnpB should not currently be considered a replacement for established CRISPR platforms, but rather a complementary compact editing architecture with unique advantages for specific biotechnology scenarios.

### 5.2 Bacterial genome editing: A foundation for platform development

Bacterial systems currently provide the strongest foundation for evaluating and engineering TnpB-based genome editing platforms. The relatively simple genome organization, efficient transformation procedures, and rapid growth characteristics of microbial hosts enable systematic investigation of fundamental design parameters, including TAM requirements, RNA scaffold function, ortholog performance, and protein engineering strategies.

Studies in model bacteria such as *Escherichia coli* have demonstrated programmable DNA targeting by TnpB systems and established principles for guide-dependent genome modification (Karvelis et al., 2021; Altae-Tran et al., 2021; Xiang et al., 2024; Tang et al., 2025). For example, SisTnpB7 supported homology-directed editing efficiencies of approximately 35%–60% across four *lacZ* targets in *E. coli* and enabled single-nucleotide editing at approximately 50% efficiency at selected sites (Feng et al., 2024). More recently, a TnpB-based editing system achieved up to 100% gene deletion efficiency in *E. coli* MG1655, whereas the same TnpB system was not functionally transferable to ATCC9637 or BL21(DE3) under the tested conditions, further illustrating the strong dependence of editing performance on host context (Tang et al., 2025). Beyond proof-of-concept editing, bacterial systems provide an important testing environment for developing optimized TnpB variants suitable for synthetic biology applications.

The compact architecture of TnpB may be particularly valuable for microbial engineering, where small genetic components facilitate plasmid construction, multiplexed pathway modification, and integration into complex synthetic circuits. Potential applications include metabolic engineering, strain optimization, and rapid functional genomics screening.

Nevertheless, bacterial validation does not necessarily predict performance in more complex organisms. Editing outcomes remain influenced by host-specific factors, including DNA repair pathways, expression compatibility, RNA stability, and target-site accessibility. Therefore, bacterial systems should be viewed as an essential engineering platform for TnpB optimization rather than evidence of universal portability.

### 5.3 Plant genome editing: compact delivery as a major opportunity

Plant genome editing represents one of the most promising application areas for TnpB because delivery constraints remain a major limitation for many genome engineering technologies (Li Q. et al., 2024; Karmakar et al., 2024; Nagalakshmi et al., 2026). The compact size of TnpB enables simplified genetic constructs and creates opportunities for transient expression, viral delivery, and potentially transgene-free editing strategies.

Recent studies have demonstrated TnpB-mediated genome editing in plant systems, including model plants and crops such as *Nicotiana benthamiana*, rice, tomato, and *Arabidopsis* (Weiss et al., 2025; Nagalakshmi et al., 2026; Li et al., 2026; Liu et al., 2026; Li Q. et al., 2024; Karmakar et al., 2024). In rice, *Agrobacterium*-mediated delivery of ISDra2 TnpB generated monoallelic editing in 38% (20/53) of T_0_ plants at the *HMBPP* locus, while biallelic *SLA4* editing produced albino mutant plants and the edited *HMBPP* allele was subsequently recovered as homozygous mutations in the T_1_ generation (Karmakar et al., 2024). Engineered TnpB variants and optimized delivery systems have further improved editing performance, highlighting the potential of TnpB as a compact alternative for plant biotechnology.

A particularly attractive feature of TnpB in plants is compatibility with delivery approaches that reduce dependence on stable transgene integration. This potential has now been demonstrated experimentally: tobacco rattle virus-mediated delivery of the engineered ISDra2 variant eTnpBc achieved systemic somatic editing efficiencies of up to approximately 90% in *Nicotiana benthamiana*, while up to 89% of offspring exhibited a mutant phenotype and editing efficiencies in progeny reached up to 100%, demonstrating high-efficiency, tissue-culture-free and transgene-free heritable editing (Nagalakshmi et al., 2026).

However, several challenges remain before broad agricultural deployment. Editing efficiency remains variable across species, genomic loci, and developmental contexts. In addition, successful somatic editing does not necessarily guarantee efficient heritable modification, particularly when reproductive tissues are not efficiently targeted.

Therefore, future plant applications will require standardized evaluation of editing efficiency, inheritance stability, guide RNA design principles, and delivery performance across diverse crop species.

### 5.4 Mammalian genome editing applications: Emerging opportunities and remaining challenges

Mammalian applications represent an important area for evaluating whether TnpB can overcome delivery limitations associated with larger genome editing systems (Marquart et al., 2024; Wang et al., 2024; Thornton et al., 2026; Li Z. et al., 2024). Recent studies have demonstrated that engineered TnpB systems can achieve programmable genome modification in mammalian cells and selected animal models, indicating that this compact nuclease architecture can be adapted for heterologous genome engineering applications (Marquart et al., 2024; Wang et al., 2024; Thornton et al., 2026; Li Z. et al., 2024). For example, single-AAV8 delivery of ISDra2 TnpB produced average indel frequencies of 9.5% at *Pcsk9* and 22.9% at *Rosa26* in mouse liver, with *Pcsk9* editing accompanied by an approximately 90% reduction in circulating *PCSK9* and a 50% reduction in plasma LDL levels (Wang et al., 2024). Further optimization of TnpBmax and computational ωRNA selection enabled AAV-mediated editing efficiencies of up to 75.3% in mouse liver and 65.9% in mouse brain (Marquart et al., 2024).

The primary biotechnology advantage of TnpB in mammalian systems is its small size (Marquart et al., 2024; Wang et al., 2024). Compared with large Cas nucleases, compact TnpB variants may provide improved compatibility with size-limited delivery platforms, including adeno-associated virus vectors and compact all-in-one genetic constructs. This feature is particularly relevant for *in vivo* genome editing applications where delivery efficiency remains a major bottleneck.

Despite this promise, mammalian translation requires substantial further development. Current limitations include variable editing efficiency, incomplete specificity characterization, dependence on optimized RNA scaffolds, and uncertainty regarding long-term genomic safety. In addition, chromatin accessibility, DNA repair pathway selection, and immune responses may strongly influence therapeutic outcomes.

Thus, the current role of TnpB in mammalian systems is best considered as an emerging genome engineering platform with potential future biomedical applications rather than a clinically mature editing technology.

### 5.5 Cross-system perspective: defining the niche of TnpB

Across biological systems, TnpB applications reveal a clear relationship between molecular engineering maturity and practical deployment potential. Bacterial systems currently provide the strongest environment for discovery and optimization, while plant systems highlight the unique value of compact delivery architectures. Mammalian applications demonstrate the long-term translational potential of TnpB but remain limited by challenges in efficiency, specificity, and delivery.

This progression suggests that the primary competitive advantage of TnpB is not universal superiority over existing genome editors, but its ability to occupy a distinct design space defined by minimal protein size and flexible engineering potential. The next phase of TnpB development will likely require co-optimization of nuclease architecture, ωRNA/reRNA configuration, and delivery architecture, because improvements in molecular activity alone may not overcome cellular implementation barriers. This engineering-to-application relationship is summarized in Figure 3.

**FIGURE 3:**
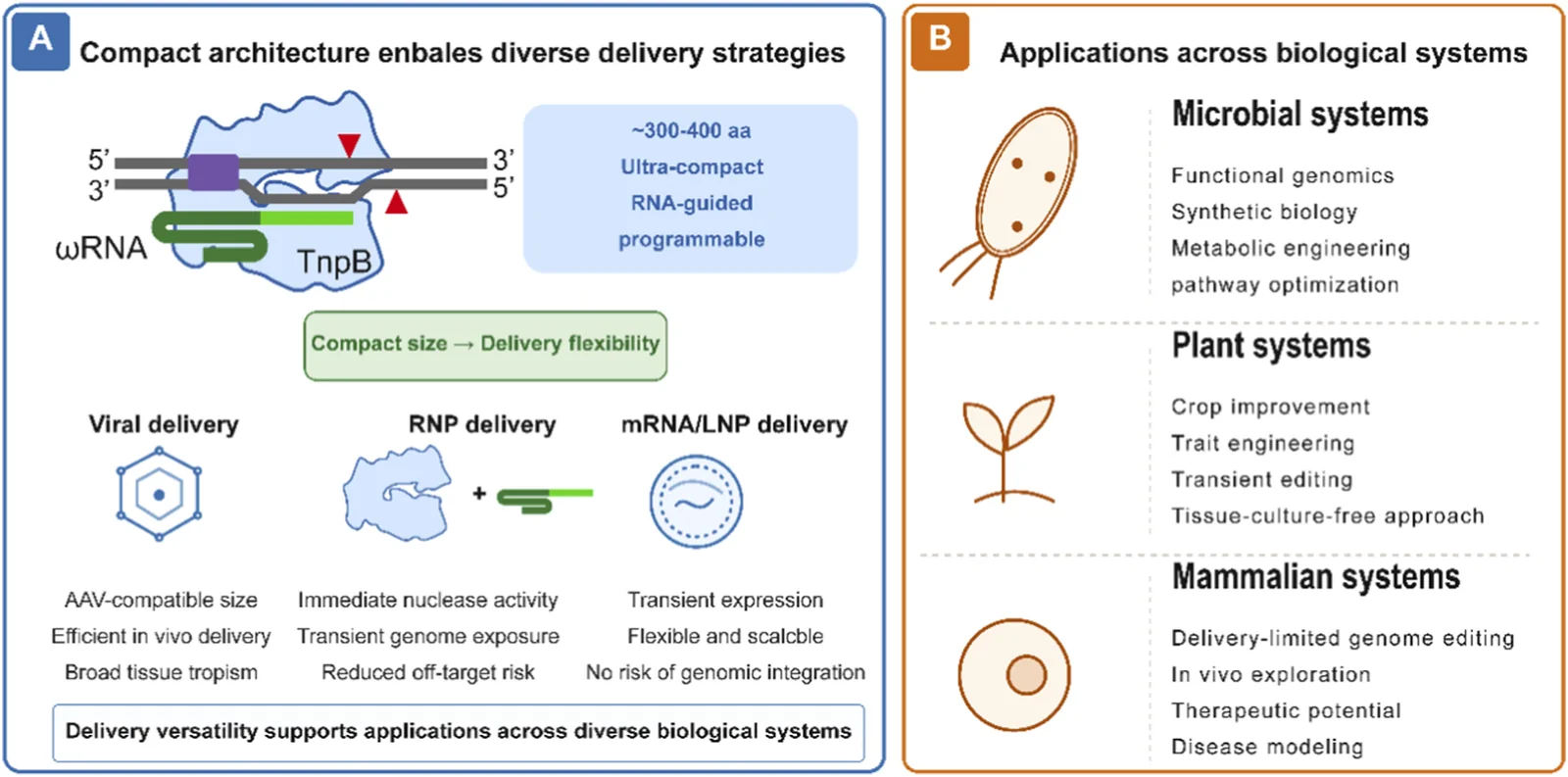
Compact architecture expands delivery flexibility and application potential of TnpB genome editors. **(A)** The small size of TnpB (∼350–400 amino acids) facilitates diverse delivery approaches, including demonstrated viral-vector and mRNA-based delivery approaches, as well as emerging RNP-based strategies. **(B)** The delivery versatility of TnpB supports applications across diverse biological systems. Microbial genome editing represents the most established application area, whereas plant and mammalian applications are emerging fields with increasing potential.

## 6 Design principles and experimentally testable roadmap for TnpB engineering

Despite rapid progress in TnpB-mediated genome editing, TnpB remains an emerging platform rather than a fully mature genome editing technology. Its compact size and RNA-guided programmability make it attractive for delivery-constrained applications and for the development of minimal genome editing systems. However, practical implementation remains limited by restrictive TAM requirements, immature ωRNA/reRNA design principles, host-dependent activity, incomplete specificity assessment, limited derivative editing modalities, and insufficient validation across biological systems. These limitations define a set of experimentally addressable design problems in which improvements should be evaluated against explicit molecular and functional benchmarks rather than as isolated gains in editing activity.

These challenges are interconnected rather than independent. For example, engineering relaxed TAM recognition may expand target accessibility but could compromise specificity. Increasing nuclease expression may improve editing efficiency but may also increase off-target cleavage. Stabilizing RNA scaffolds may enhance activity but could reduce guide flexibility or alter mismatch tolerance. Therefore, future development of TnpB-based editors will require integrated optimization of protein architecture, ωRNA/reRNA configuration, target range, specificity, expression, and delivery rather than isolated improvement of individual parameters. Each proposed improvement should therefore be tested not only for its intended effect but also for predictable consequences on coupled properties—for example, whether TAM expansion alters off-target discrimination, whether RNA-scaffold optimization remains beneficial across protein variants, and whether increased expression improves on-target editing without disproportionately increasing nuclease exposure or off-target activity.

### 6.1 Expanding TAM compatibility without compromising specificity

A central limitation of TnpB-based genome editing is its ortholog-specific TAM dependence, which restricts target-site accessibility and remains less systematically mapped than the PAM rules of mature Cas9 and Cas12 systems. Recent studies nevertheless demonstrate that this constraint is engineerable: structure-guided substitution of the ISDra2 TAM-interacting residue K76 broadened recognition toward alternative TAMs, whereas naturally flexible TAM recognition by SisTnpB7 showed that weaker TAMs can support efficient editing while maintaining strong target discrimination (Marquart et al., 2024; Feng et al., 2024). Accordingly, a practical design principle is to combine natural ortholog selection, high-throughput TAM profiling, and structure-guided engineering of TAM-interacting residues to expand target range while preserving predictable motif recognition. Importantly, TAM relaxation should be paired with genome-wide specificity profiling, because expanded motif tolerance may weaken target discrimination and increase off-target risk. An engineered TnpB variant should therefore be considered improved only when broader TAM compatibility increases the accessible target space without a disproportionate loss of on-target activity or target discrimination under matched expression conditions.

### 6.2 Establishing predictive ωRNA/reRNA design rules

The ωRNA/reRNA scaffold remains a second major source of unpredictability because it functions both as a programmable guide and as a structural component required for TnpB ribonucleoprotein assembly. Recent large-scale guide profiling and reRNA mutational scanning have begun to define sequence- and structure-dependent activity rules for ISDra2 TnpB, but their transferability across orthologs, engineered protein variants, and host systems remains insufficiently established (Marquart et al., 2024; Thornton et al., 2026). A practical design principle is therefore to preserve structurally constrained scaffold elements required for RNP assembly while systematically testing modifications in flexible regions and guide sequences for gains in activity. Importantly, RNA variants should not be optimized independently of the protein component, because beneficial reRNA mutations can become neutral or detrimental when paired with independently engineered TnpB variants (Thornton et al., 2026). An improved ωRNA/reRNA design should therefore demonstrate reproducible gains across multiple target sites and retain its benefit when tested with the intended TnpB protein and host context, rather than being selected solely from performance at a single locus or in a single screening system.

### 6.3 Improving host transferability and cross-system benchmarking

TnpB activity cannot be inferred from biochemical cleavage or from validation in a single host, because editing outcomes are shaped by codon usage, expression level, RNA stability, localization, chromatin accessibility, DNA repair, and delivery efficiency. Standardized cross-system benchmarking should therefore test candidate orthologs and engineered variants across comparable targets, guide designs, expression formats, and delivery conditions in bacterial, plant, and mammalian systems (Cao et al., 2025; Xin et al., 2026). Reporting should include target sequence, TAM context, RNA scaffold design, delivery method, editing efficiency, indel spectrum, and off-target assessment to enable meaningful comparison across studies. A TnpB variant should therefore be considered broadly transferable only when its performance advantage is reproduced across multiple loci and host contexts under matched or explicitly normalized experimental conditions, rather than inferred from high activity in a single assay or organism.

### 6.4 Optimizing delivery while controlling nuclease exposure

The compact size of TnpB is a major advantage for size-restricted delivery, including AAV-compatible and all-in-one vector designs, but delivery efficiency still depends on promoter choice, codon usage, nuclear localization, RNA expression cassette design, vector format, tissue tropism, and expression duration. Future delivery strategies should optimize these parameters together with nuclease exposure, because prolonged expression may increase editing frequency but also elevate off-target risk. For TnpB, both mRNA/ωRNA delivery and single-AAV delivery have already been demonstrated experimentally, including embryo editing and efficient somatic editing in mouse tissues (Li Z. et al., 2024; Marquart et al., 2024), whereas direct delivery of purified TnpB ribonucleoproteins and nanoparticle-based formulations remains comparatively underexplored. A practical design principle is therefore to match the delivery format to the required duration and anatomical context of editing rather than assuming that maximal or prolonged nuclease expression is always advantageous. Delivery strategies should be benchmarked by jointly measuring on-target editing, duration of nuclease exposure, tissue or cell-type coverage, and off-target activity under matched dosing conditions.

### 6.5 Defining genome-wide specificity and safety profiles

Initial targeted and genome-wide analyses have generally indicated favorable specificity for several TnpB systems, but standardized specificity benchmarks across orthologs, engineered variants, guides, and delivery conditions remain limited. Off-target activity should be assessed for both native and engineered variants using unbiased genome-wide detection methods under realistic delivery and expression conditions, because TAM compatibility, guide mismatch tolerance, RNA scaffold structure, nuclease abundance, and exposure duration can all influence fidelity. This comparison is particularly important for activity-enhancing variants, because increased on-target activity can be accompanied by increased off-target editing at some mismatched sites, as demonstrated for engineered ISDra2 variants in human cells (Thornton et al., 2026). For therapeutic or agricultural applications, safety profiling should also examine large deletions, chromosomal rearrangements, unintended donor integration, transcriptomic perturbation, immune responses, and long-term stability of edited cells or organisms. A practical benchmark should therefore require specificity to be evaluated together with on-target activity under matched expression and delivery conditions, rather than comparing off-target frequencies independently across experiments.

### 6.6 Developing derivative TnpB editing modalities

Although nuclease-mediated cleavage and indel generation remain the dominant applications of TnpB, derivative modalities have begun to emerge. TnpB-based base editing has been demonstrated in *Streptomyces*, while engineered catalytically inactive TnpB systems have enabled transcriptional activation and adenine base editing in mammalian cells (Luo et al., 2025; Lu et al., 2026). For example, the STAGE platform enabled single and multiplexed C·G-to-T·A base editing with efficiencies exceeding 75%, whereas the enTnpB platform supported transcriptional activation and A-to-G base editing in mammalian cells, with adenine conversion reaching 16.2% at the best-performing tested locus. Further development should therefore focus on optimized fusion architectures, compatible linker designs, preservation of protein–RNA target recognition, and control of editing windows under TAM constraints. These modalities remain less mature than Cas9-and Cas12-derived editing platforms. Their broader utility should be evaluated by whether fusion or catalytic modification preserves guide compatibility, TAM-constrained targeting range, editing or regulatory activity, specificity, and delivery efficiency across multiple target sites.

### 6.7 Toward an expanded coupled-constraint design space

Future TnpB development should aim to expand the permissive activity regime while controlling fidelity. A practical roadmap should combine ortholog discovery, high-throughput TAM and guide profiling, structure-guided protein–RNA co-engineering, standardized cross-host benchmarking, genome-wide specificity assessment, and derivative editor development. These efforts should be pursued together because target range, activity, delivery efficiency, and fidelity are interdependent rather than independently optimizable traits. Accordingly, candidate TnpB systems should be advanced only when improvements remain reproducible across multiple targets and are evaluated simultaneously for activity, TAM compatibility, RNA-scaffold performance, specificity, host transferability, and delivery feasibility. This integrated benchmarking framework provides an experimentally testable route for determining whether an engineered TnpB variant expands the functional design space without introducing unacceptable trade-offs in another constraint layer.

## 7 Conclusion

TnpB represents a compact RNA-guided nuclease platform derived from IS200/IS605-family transposable elements. Its editing performance is determined not only by nuclease activity but also by coordinated interactions among protein architecture, RNA scaffold organization, TAM recognition, and target DNA engagement.

This review highlights that engineering TnpB requires a coupled optimization framework integrating protein engineering, RNA scaffold design, target recognition, expression optimization, and delivery strategies. Rather than replacing established CRISPR-Cas systems, TnpB provides complementary opportunities for compact genome editing while presenting challenges associated with ortholog diversity, context-dependent activity, and incomplete design rules.

Future advances will benefit from predictive design principles that connect molecular constraints with editing outcomes, enabling the development of more efficient, specific, and programmable TnpB-based technologies.
